# Developmental trajectories of outpatient mental health service contact from childhood to early adulthood in an Australian birth cohort

**DOI:** 10.3389/fpsyt.2026.1635802

**Published:** 2026-02-13

**Authors:** James M. Ogilvie, Belinda Crissman, Emily Hurren, Carleen M. Thompson, Aydan Kuluk, Troy Allard, Lisa Broidy, Susan Dennison, Steve Kisely, Anna Stewart

**Affiliations:** 1 Griffith Criminology Institute, Griffith University, Brisbane, QLD, Australia; 2 School of Criminology and Criminal Justice, Griffith University, Brisbane, QLD, Australia; 3 Department of Sociology, University of New Mexico, Albuquerque, NM, United States; 4 School of Medicine, The University of Queensland, Brisbane, QLD, Australia

**Keywords:** administrative data, birth cohort, community mental health, psychosis, trajectory modelling

## Abstract

**Background:**

Despite increasing empirical support for primary intervention in the early development of mental disorders, limited lifespan developmental research exists to identify the potential timing of such interventions. This study takes a novel approach by applying Group Based Trajectory Modelling (GBTM) to identify patterns of outpatient mental health service (MHS) contacts over the early life-course. GBTM is a statistical method that can assist in identifying subgroups in a population that follow similar developmental trajectories over time.

**Methods:**

We analyzed longitudinal (age ≈10 to 23 years) outpatient MHS contact data for an Australian birth cohort (N = 5,359) using GBTM. We examined variation across trajectory groups by sex, Indigenous status (non-Indigenous/Indigenous Australians), psychiatric diagnoses associated with hospital admissions, place of residence (metropolitan, regional, remote), and socioeconomic status.

**Results:**

Four distinct trajectories of MHS contacts were identified, including escalating (5.8%), low (64.5%), adolescent limited (14.5%), and childhood peak declining (15.3%) trajectories. The escalating contacts trajectory group contained the fewest individuals (n = 308) but accounted for the highest proportion of contacts (32.3%), providing evidence of the need for and potential value of early identification and intervention for these individuals. Variations were also noted across trajectory groups for place of residence, socioeconomic status, and diagnosed mental disorders from hospital admissions.

**Conclusions:**

GBTM can assist examination of variations in MHS contacts across the life course. Efficacious intervention with a small but distinct group of vulnerable individuals may meaningfully impact available system resources and improve their outcomes.

## Introduction

The importance of early intervention to the prevention and amelioration of mental disorder symptomatology over the life course is well-recognized in the clinical literature (1). However, the task of identifying key services and timing of interventions is challenging due to differences in mental disorder progression, service use and availability, and differences across age, race/ethnicity and gender (2). There are three main challenges faced when trying to target key populations for early intervention. First, mental disorder and service needs vary across the life-course of individuals. Different disorders have different ages of onset (3), and a formal mental disorder diagnosis is often preceded by an “at risk mental state” earlier in the developmental life course wherein the individual experiences low-intensity symptoms (2). Empirical evidence indicates that there is a progression from sub-diagnostic symptoms to mental disorders including psychosis, persistent mood, anxiety, personality and substance use disorders (4, 5). Several studies have recommended the use of clinical staging models aimed at improving benefits by addressing specific needs at specific stages of mental disorder development (e.g., 2, 6). Clinical staging approaches have the potential to improve mental health outcomes and ameliorate the disease burden and costs associated with mental disorder. However, there is limited extant lifespan developmental research indicating the timing of interventions in relation to mental health service (MHS) contacts. To implement such a strategy, key time points, vulnerable populations, and services must first be identified.

Second, social and individual factors can impact on service access and engagement. For example, not all individuals who experience mental disorder will seek MHSs (7). Research utilizing a sample of Australian adolescents indicated that most individuals with a mental disorder sought services, however higher rates of service seeking were observed among those with suicidality, female sex, older age (15–17 years), disadvantaged families, history of substance abuse, and multiple mental disorders (7). Self-reported MHS use within a rural Australian community also highlighted variations in service utilization, including higher rates of help-seeking among individuals without a partner, and those with more severe mental health problems, greater adversity, and poorer finances (8).

A third challenge is that in Australia there is also considerable complexity associated with racial differences in diagnosed mental disorder and MHS engagement (9). Australia’s history of forced colonization and associated intergenerational trauma, as well as systemic and institutional factors, have been linked to overrepresentation of mental disorder among Indigenous Australians (Aboriginal and Torres Strait Islander peoples) (10). Additionally, Indigenous Australians may experience additional barriers to MHS use, particularly when living in rural and remote areas (10). Australian data has indicated a higher treated prevalence of psychotic disorders in Indigenous men and women compared to national prevalence rates, (11). Gynther et al. (11) suggested that environmental and neurodevelopmental factors likely play an etiological role in psychosis, and that social factors contribute to vulnerability and mortality.

Administrative data specifying contacts with MHSs, and recorded diagnoses of mental disorder, are a valuable tool for research on mental health across the life-course. Administrative data can reveal heterogeneity in mental disorder prevalence across population subgroups, as well as variations in MHS accessibility, provision, and engagement over time and place. Each of these knowledge points is crucial to understanding risks and needs of individuals and subgroups within a population as well as intervention points and necessary resourcing. Administrative population-based cohort data have been used internationally to analyses relationships and identify vulnerabilities with regards to mental disorder. For example, Swedish administrative data was used to examine the relationship between substance use and other psychiatric disorders with regards to premature mortality and community service orders (12). Administrative cohort data have also been used to identify the prevalence of psychiatric disorders for Indigenous Australians (13).

It is essential therefore to identify heterogeneity in life-course patterns of MHS contacts and mental disorder diagnoses, with the aim of establishing timepoints and resourcing criteria for interventions. One method of identifying life course patterns is group-based trajectory modelling (GBTM), which identifies clusters of individuals following a similar developmental trajectory on an outcome of interest (14). GBTM has been applied in clinical research, including mapping the developmental course of one or more clinical trajectories over time, identifying predictors of trajectory group membership, and evaluating the impact of turning-point events or therapeutic interventions (14). For example, GBTM has been used to model medication adherence patterns of hospitalization for coronary heart disease (15), and patterns of emergency department use and hospitalization among individuals with a functional disability (16). However, GBTM has not (to the authors’ knowledge) been applied to life course analysis relating to MHS contacts.

In the current study, we extend the use of GBTM beyond its previous applications in clinical research, to apply GBTM to produce life-course trajectories of MHS contact, to explore variations in service access with consideration of age/timing, types of diagnosed mental disorder, sex, Indigenous status, SES, and place of residence. To our knowledge, this will be the first time this method has been applied to long-term MHS contact patterns. This knowledge can be used to identify specific groups that may respond differently to interventions, as well as developmental timepoints and key services to target treatment most efficaciously. Specifically, we apply GBTM to longitudinal outpatient MHS administrative data from an Australian birth cohort. A large longitudinal administrative dataset allows us to address two main aims:

1. Utilize GBTM to explore patterns of MHS contacts across the life-course (to early adulthood) of a birth cohort from Queensland, Australia. Within this aim there are two research questions: a) are there distinct trajectories based on timing and frequency of MHS contacts? and b) is there variation in relation to distribution of sex and Indigenous status, SES, place of residence, and mental disorder diagnoses across these trajectories?
2. Demonstrate the utility of GBTM as technique to examine variations in MHS contacts across the life course, and by extension, to act as an exemplar for future studies in life-course research perspectives of mental disorder and its associated developmental outcomes.

## Methods

### Data sources and sample

This study draws data from the Queensland Cross-sector Research Collaboration (QCRC) birth cohorts (see 17). We utilize longitudinal administrative records across the following government agencies/systems: Queensland Registry of Births, Deaths and Marriages (births and deaths records); and Queensland Health (hospital admissions associated with mental health diagnoses and outpatient MHS records). Data are stored in Griffith University’s Social Analytics Lab, which is a secure purpose-built facility for storing sensitive data. The study was approved by the Griffith University Human Research Ethics Committee (HREC 2010/479).

The study draws on a population-based birth cohort comprising all individuals born in Queensland in 1990 (N = 45,422), of whom 5,359 (11.8%) had at least one outpatient MHS contact up to age 24 years (demographic details provided in **Table 1**).

**Table 1:** Outpatient MHS contacts up to age 24 years by sex and Indigenous status.

Demographic group	Total cohort		MHS contact	
	N	%	N	%
Indigenous females	1,280	2.8	386	7.2
Indigenous males	1,589	3.5	459	8.6
Non-Indigenous females	20,759	45.7	2,315	43.2
Non-Indigenous males	21,794	48.0	2,199	41.0
Total	45,422	100.0	5,359	100.0

The sample for the study was everyone who had experienced an outpatient MHS contact (50.4% female; 15.8% Indigenous). Female, χ^2^(1) = 8.52, *p<*0.01, Cramer’s V (φ_c_) = .01, and Indigenous, χ^2^(1) = 915.43, *p<*0.001, φ_c_ = .14, individuals were significantly overrepresented in this sample compared to male and non-Indigenous individuals, respectively. By 24 years of age, outpatient MHS contact was identified for 30.16% of Indigenous females, 28.89% of Indigenous males, 11.15% of non-Indigenous females, and 10.09% of non-Indigenous males.

### Outpatient MHS contacts

Details about MHS contacts were derived from the Queensland Health Consumer Integrated Mental Health Application (CIMHA) dataset. CIMHA covers information on contacts with outpatient Community MHSs (CMHS). Contacts recorded in CIMHA relate to service events delivered by healthcare providers, which could include in-person contacts (e.g., individual and group treatment sessions), telephone contacts (both with consumer and other service providers), emails, consultation, and assessment activities. CIMHA recorded contacts relate to healthcare provision activities, and therefore act as a proxy for the intensity of service utilization for consumers. The number of service contacts recorded in CIMHA was summed for each calendar year in the observation period for each individual. CIMHA data cover the period from September 2000 to December 2013. Therefore, information relating to MHS usage were available from about age ≈ 10 to 23 years for the 1990 cohort. Although some diagnostic information is recorded in CIMHA, it was not used to establish the presence of psychiatric diagnoses on the advice of data custodians that this information was not consistently or reliably coded.

### Covariates

*Indigenous status* was assigned if an individual had ever identified as Indigenous Australian^1^ in any of the QCRC databases, which is consistent with best-practice guidelines for linked data (18). *Sex* was assigned based on the balance of probabilities for all QCRC databases. *Place of residence* at first contact was coded according to the Australian Standard Geographical Classification – Remoteness Area classification (19); with three classes^2^ (metropolitan; inner/outer regional; and remote/very remote). *Socioeconomic status* was measured based on the *Index of Relative Socio-economic Advantage and Disadvantage* (IRSAD) from the Socio-Economic Indexes for Areas (20) and was coded for place of residence at first contact. IRSAD ranges from 1 to 10, where lower scores indicate greater disadvantage and lack of advantage, and higher scores indicate a relative lack of disadvantage and greater advantage. We used a binary measure of whether a person’s location of residence at first contact was characterized by the lowest IRSAD value of 1.

*Age at first MHS contact* was determined by the date of first recorded CIMHA contact. *Psychiatric diagnoses* associated with hospital admissions for the sample (of individuals with at least one CIMHA contact) were extracted from the Queensland Hospital Admitted Patient Data Collection (QHAPDC). This dataset contains information for public and private hospital admissions in Queensland, including dates of admission and diagnostic information associated with these admissions coded according to the International Statistical Classification of Diseases and Related Health Problems – tenth edition Australian modification (ICD-10AM; 21). We coded the presence of any psychiatric disorder (ICD-10 *Mental and behavioral disorders* [codes F00 to F99], as well as suicidal ideation [code R45.8] and self-harm [codes X60 through X84]). In addition, we coded individuals for the presence or absence of three specific diagnostic classes: psychotic mental disorders (schizophrenia, schizotypal and delusional disorders [F20-F29], severe or psychotic affective disorders [F30, F31, F32.2, F32.3], and psychotic disorders related to substance use [F10.5, F11.5, F12.5, F13.5, F14.5, F15.5, F15.50, F15.51, F15.59, F15.70, F16.5, F17.5, F18.5, F19.5, F19.7]); mood and anxiety disorders [F32.0, F32.1, F32.8, F32.9, F33-F48]; and substance use disorders excluding those involving psychosis [F10-F19]. We selected mood and anxiety, and substance use disorders as the most commonly occurring diagnoses in the cohort based on previous research (13). Psychotic disorders were selected, since these conditions are associated with significant long-term mental health service usage (22).

### Analytical strategy

We first describe MHS contacts across sex and Indigenous status. We then estimate longitudinal patterns of MHS contacts using GBTM (23–25). GBTM is a specialized application of finite mixture modelling used to identify clusters of individuals following similar progressions of behaviors or outcomes longitudinally. The trajectory analyses are reported in line with the Guidelines for Reporting on Latent Trajectory Studies checklist (26).

Trajectory models were fit using the *FLXMRglm* driver in the *flexmix* package (version 2.3-17; 27) for R (version 4.2.0; 28). A Poisson model using a quadratic function of age/time was adopted given they are appropriate to model count variables characterized by a low minimum value and an unbound maximum. Service contacts were significantly positively skewed across the observation period (i.e., most individuals only have a few service contacts recorded). To estimate trajectory models, we capped (i.e., top-coded) service contacts at a maximum of 100 contacts per year for each individual to minimize skew and the influence of outliers on the model, and to ensure model convergence. Models were fit for one to six classes, with the decision to not test beyond six classes made to preserve parsimony and interpretability and ensure that class sizes were large enough to provide adequate statistical power for subsequent analyses. Model selection was guided by multiple goodness-of-fit and classification accuracy statistics, including log likelihood, Akaike information criterion (AIC), Bayesian information criterion (BIC), entropy (29), the average posterior probability (AvePP) of group classification for most likely group membership, and odds of correct classification (OCC; 30). The best fitting models are typically identified by low log likelihood, AIC and BIC values (31), and entropy and AvePP values closest to one. AvePPs above 0.70 and OCCs greater than 5 for all groups are considered indicative of adequate fit (30). Consideration was also given to the interpretability of the estimated models and the principle of parsimony, referring to the preference for selecting the model with the fewest number of classes that is statistically significant and substantively adequate (32, 33). Individuals were assigned to trajectory groups based on maximum posterior group probabilities, with group membership used as dependent variables for subsequent analyses. This approach was appropriate given the high entropy values (>.80) of the fitted models, which indicate high separation between the modelled groups (34).

Potential correlates of group membership were analyzed descriptively using chi-square tests for categorical variables (i.e., sex and Indigenous status, place of residence, disorder prevalence) and ANOVA for continuous variables (i.e., age at first contact, mean contacts, IRSAD).

## Results

### Outpatient contacts and disorders

We first describe patterns of outpatient MHS contacts and compare contacts across sex and Indigenous status for the full observation period (**Table 2**). The sample was responsible for 136,507 MHS contacts during the observation period, with a mean of 25.47 contacts per individual. However, there was wide variation in the number of contacts per individual (SD = 54.04), with the median number of contacts per individual being 10. There was no significant difference in mean MHS contacts across demographic groups defined by the intersection of sex and Indigenous status, *F*(3,5355) = 1.40, *p* = .24, η^2^ = .001. There was a small but significant difference in mean age at first contact across demographic groups, *F*(3,5355) = 12.16, *p* <.001, η^2^ = .01, with non-Indigenous males significantly younger at first contact compared to both Indigenous and non-Indigenous females using Tukey multiple comparisons of means adjustment.

**Table 2:** Descriptive information for MHS contacts, diagnosed disorder presence, and covariates by sex and Indigenous status (*n* = 5,359).

Variable	Demographic					Group difference
	Indigenous females	Indigenous males	Non-Indigenous females	Non-Indigenous males	Total	χ^2†^ (φ_c_)/ F (Ƞ^2^)
*Outpatient MHS contacts*						
n (% of total sample with MH service contact)	386 (7.2%)	459 (8.6%)	2,315 (43.2%)	2,199 (41.0%)	5,359 (100.0%)	
Total contacts [n, (% of total contacts for sample)]	11,286 (8.3%)	13,108 (9.6%)	58,331 (42.7%)	53,782 (39.4%)	136,507 (100.0%)	
Mean contacts per person [M, (SD)]	29.24 (75.80)	28.56 (57.95)	25.20 (57.23)	24.46 (44.28)	25.47 (54.04)	1.40^b^ (>.01)
Age at first contact [M, (SD)]	16.30 (3.33)	15.77 (3.74)	15.93 (3.22)	15.40 (3.88)	15.73 (3.57)	12.16***^b^ (.01)
Hospital-based diagnosed mental disorder prevalence^c^						
Any disorder	155 (40.2%)	190 (41.4%)	790 (34.1%)	736 (33.5%)	1,871 (34.9%)	15.80** (.05)
Psychotic mental disorder	26 (7.5%)	50 (10.9%)	192 (8.3%)	197 (9.0%)	468 (8.7%)	4.11 (.03)
Mood and anxiety disorder	86 (22.3%)	61 (13.3%)	506 (21.9%)	351 (16.0%)	1,004 (18.7%)	38.06*** (.08)
Substance use disorder	87 (22.5%)	125 (27.2%)	232 (10.0%)	351 (16.0%)	795 (14.8%)	118.64*** (.15)
Place of residence						
Missing [n, (%)]	22 (5.7%)	49 (10.7%)	143 (6.2%)	296 (13.5%)	510 (9.5%)	76.95*** (.12)
Metropolitan^a^ [n, (%)]	118 (30.6%)	148 (32.2%)	1,173 (50.7%)	1,000 (45.5%)	2,439 (45.5%)	92.17*** (.13)
Regional^a^ [n, (%)]	194 (50.3%)	213 (46.4%)	942 (40.7%)	863 (39.3%)	2,212 (41.3%)	21.90*** (.06)
Remote/very remote^a^ [n, (%)]	52 (13.5%)	49 (10.7%)	57 (2.5%)	40 (1.8%)	198 (3.7%)	198.18*** (.19)
*SES^c^*						
Lowest IRSAD^d^ [n, (%)]	67 (18.4%)	109 (26.3%)	201 (9.2%)	204 (10.7%)	581 (12.0%)	113.02*** (.15)

*p <.05, **p <.01, ***p <.001. IRSAD, index of relative socioeconomic advantage and disadvantage; SES, socioeconomic status.

^†^Pearson’s chi-squared test (*df* = 1) with Yates’ continuity correction; φc = Cramer’s V effect size for chi-squared test.

a Excludes cases with missing data.

b ANOVA F-test df = 3/5175.

c Derived from hospital admissions.

d Missing data reported for *Place of residence* also applies to SES since IRSAD is calculated from location.

We examined the prevalence of any and specific mental disorders diagnosed from hospital admissions for the sample of individuals with at least one community MHS contact: 1,871 (34.9%) had received any mental disorder diagnosis; 468 (8.7%) had a psychotic mental disorder; 1,004 (18.7%) had a mood or anxiety disorder; and 795 (14.8%) had a substance use disorder. There was a significant but small difference across demographic groups for the prevalence of any mental disorder diagnosis, χ^2^ (3, *N* = 5359) = 15.80, *p<*0.01, φ_c_ = .05. *Post-hoc* analysis of chi-square residuals indicated that Indigenous males were more likely to be diagnosed with any mental disorder. There was a significant difference across demographic groups for the prevalence of mood and anxiety disorders, χ^2^ (3, *N* = 5359) = 38.06, *p<*0.001, φ_c_ = .08. Indigenous and non-Indigenous females were more likely to be diagnosed with mood and anxiety disorders, while Indigenous and non-Indigenous males were less likely to diagnosed with these disorders (based on *post-hoc* analysis of chi-square residuals). There was a significant difference across demographic groups for the prevalence of substance use disorders, χ^2^ (3, *N* = 5359) = 118.64, *p<*0.001, φ_c_ = .15. Indigenous males and females were more likely to be diagnosed with a substance use disorder, but non-Indigenous females were less likely to be diagnosed with these disorders (based on *post-hoc* analysis of chi-square residuals). There was no difference across demographic groups in the prevalence of diagnosed psychotic disorders.

We also examined the distribution of demographic groups across place of residence, including those who resided in locations of greatest socioeconomic disadvantage (see **Table 2** for details). Non-Indigenous people were more likely to reside in metropolitan areas, and Indigenous people were more likely to reside in regional and remote/very remote areas, which corresponded with areas of greatest socioeconomic disadvantage.

**Figure 1:**
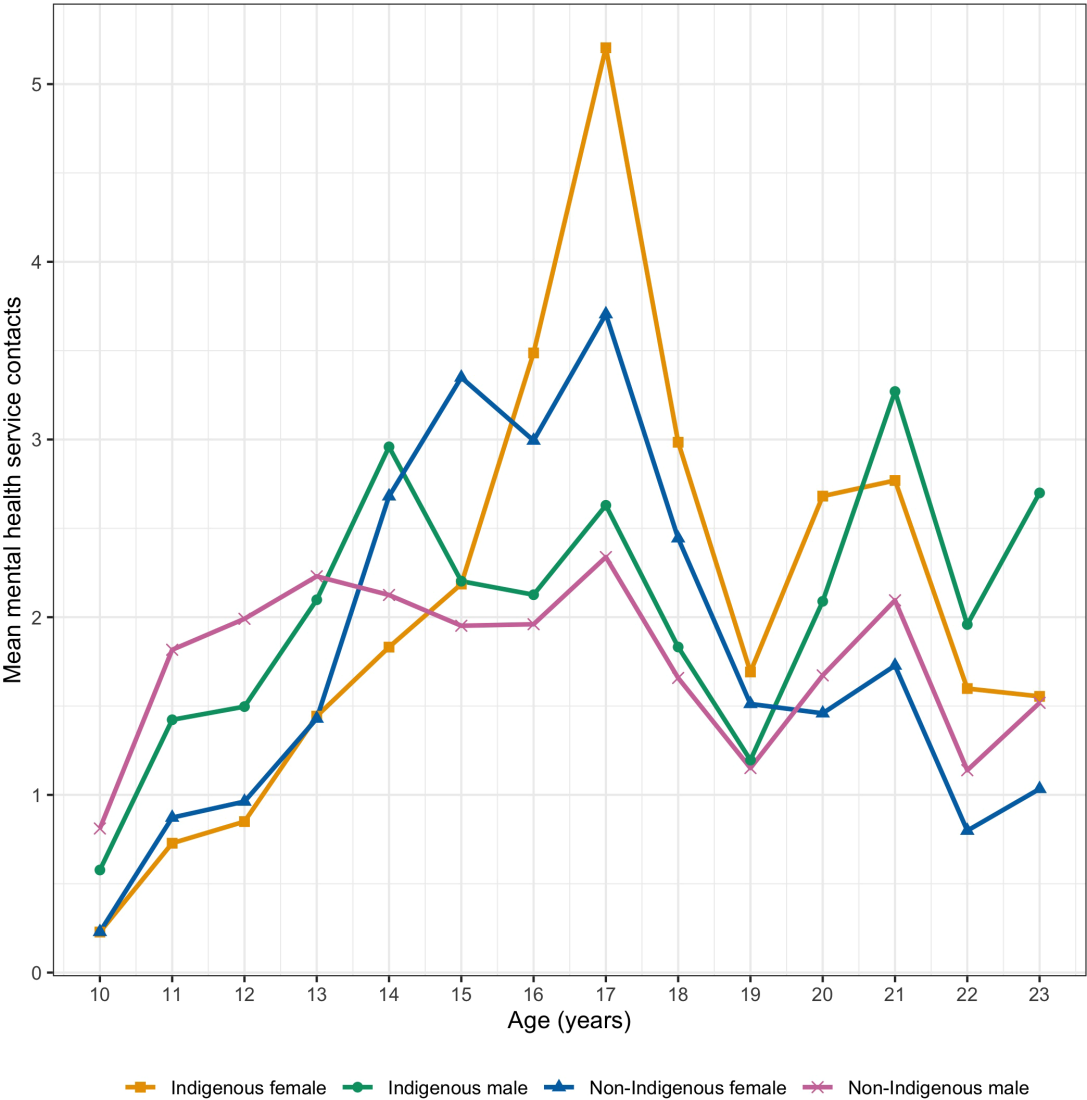
Mean counts of outpatient MHS contacts by age, stratified by sex and Indigenous status.

Mean counts of MHS contacts were plotted by age and stratified by the intersection of sex and Indigenous status (**Figure 1**) and show two prominent peaks at 17 and 21 years. There was a dip in mean service contacts between these years, which could reflect a transition between youth and adult MHSs. Indigenous females exhibited the highest mean service contacts at the first most prominent peak at 17 years, while Indigenous males exhibited the highest mean contacts at the second peak at 21 years. These patterns demonstrate aggregate age-graded differences in MHS contacts that varied across both Indigenous status and sex.

### MHS trajectories

Goodness-of-fit and classification accuracy statistics for the GBTM model selection process are provided in **Table 3**. AIC and BIC values continued to improve with each additional group included in the model. However, the addition of groups beyond four resulted in increasingly small groups (< 3.0% group membership), which suggested poor model convergence and model collapse (33). The four-group model produced distinct groups and interpretable patterns of MHS contact trajectories. Therefore, a four-group trajectory solution was selected as the best model for MHS contacts based on goodness-of-fit (AIC and BIC) classification statistics (average posterior probability, odds of correct classification) and the interpretability of trajectory shapes.

**Table 3:** Model selection goodness-of-fit and classification accuracy statistics for one to six group models of MHS contacts.

Groups	Log-likelihood	AIC	BIC	Entropy	AvePP	OCC	Group membership *n* (%)
1	-337,839.8	675,685.6	675,713.3	–	1	–	5,359 (100.0%)
2	-265,152.8	530,319.5	530,384.1	.99	.99;.99	676.78; 114.59	821 (15.3%); 4,538 (84.7%)
3	-232,036.2	464,094.4	464,195.9	.98	.99;.99;.99	323.57; 1,854.77; 75.53	1,215 (22.7%); 395 (7.4%); 3,749 (70.0%)
**4**	**-215,567.8**	**431,165.6**	**431303.9**	**.98**	**.99;.99;.98;.99**	**3,628.54; 80.48; 291.70; 518.63**	**308 (5.8%); 3,454 (64.5%); 776 (14.5%); 821 (15.3%)**
5	-202,797.3	405,632.6	405,807.9	.98	.98;.99;.99;.99;.98	318.48; 20,944.10; 685.62; 81.96; 399.52	729 (13.6%); 138 (2.6%); 549 (10.2%); 3,195 (59.6%); 748 (14.0%)
6	-193,976.3	387,998.6	388,210.8	.98	.99;.98;.99;.98;.96;.98	12,658,163.78; 819.60; 73.29; 600.90; 104.40; 441.17	199 (2.2%); 372 (6.9%); 2,648 (49.4%); 464 (8.7%); 1,068 (19.9%); 688 (12.8%)

AvePP, Average posterior probability of group classification for most likely group membership; OCC, Odds of correct classification; ‘-’, not applicable due to single group. Selected model in bold.

**Figure 2:**
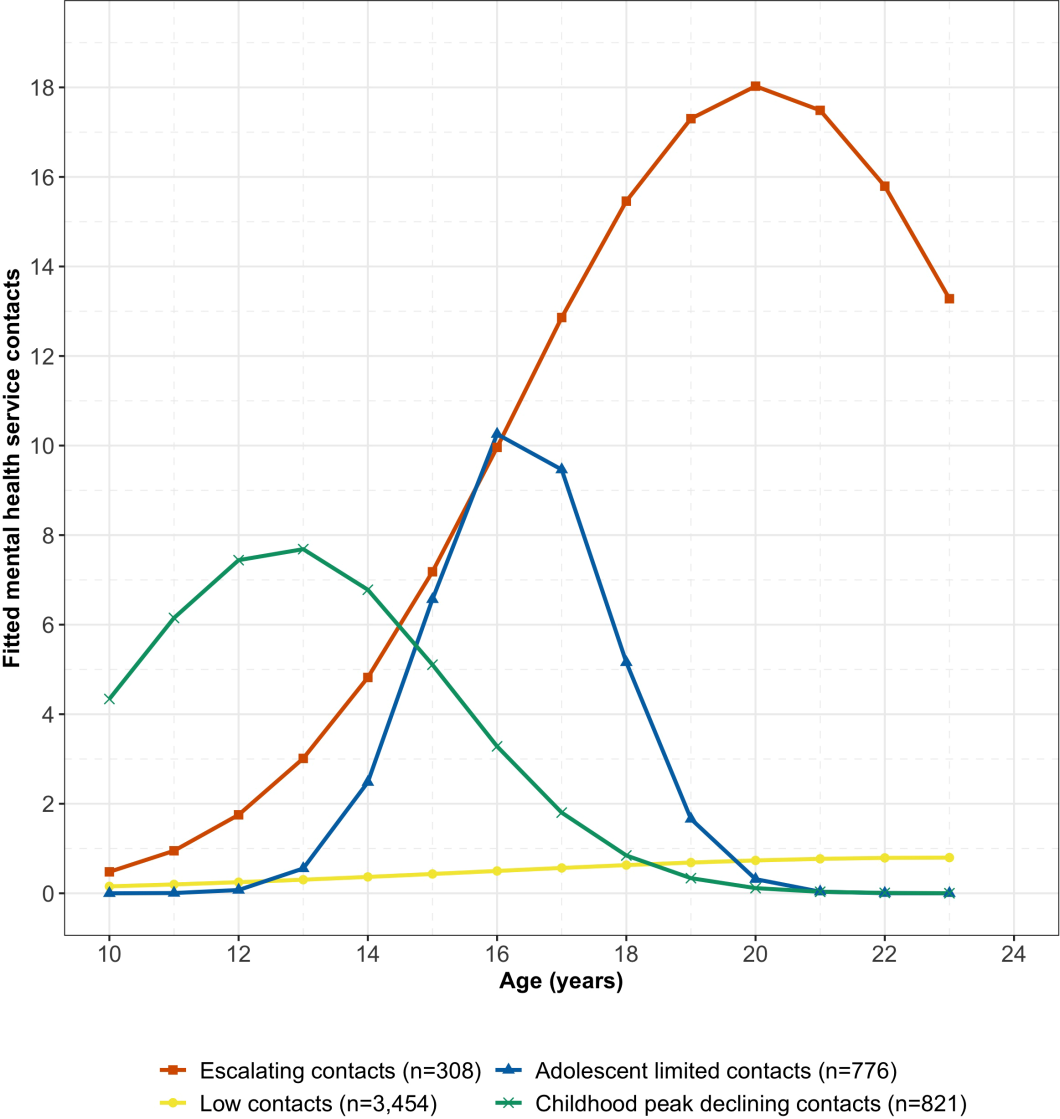
Fitted mean MHS contact counts by age for the four-group trajectory model.

**Table 4** details model coefficients, classification statistics and descriptive information for the four-group trajectory model and **Figure 2** illustrates aggregated trajectory patterns of fitted mean MHS contact counts by age for each group. The four-group trajectory model was interpreted to consist of escalating (*n* = 308, 5.8%), low (*n* = 3,454, 64.5%), adolescent limited (*n* = 776, 14.5%) and childhood peak declining (*n* = 821, 15.3%) MHS contacts groups. The low contacts group contained the majority (64.5%) of individuals in the sample, but only accounted for 18.8% of all service contacts. In contrast, the escalating contacts group contained the fewest individuals (5.8%) but accounted for the highest proportion of contacts (32.3%), demonstrating that service contacts were concentrated within a comparatively small group of individuals.

**Table 4:** Four group MHS contacts trajectory model coefficients, classification diagnostic statistics and descriptive information.

Variable	Group				Group difference
	1	2	3	4	
	Escalating contacts	Low contacts	Adolescent limited contacts	Childhood peak declining contacts	χ^2†^ (φ_c_)/ F (Ƞ2)
N individuals	308	3,454	776	821	−
% sample	5.8%	64.5%	14.5%	15.3%	−
Coefficients					
Intercept	-11.51	-5.39	-67.98	-10.71	−
Age	1.44	0.45	8.61	2.01	−
Age^2^	-0.04	-0.01	-0.26	-0.08	−
Classification					
AvePP	.99	.99	.98	.99	−
OCC	3,628.54	80.48	291.70	518.63	−
Predicted proportion	5.7%	64.4%	14.4%	15.4%	−
Demographic^a^					155.20*** (.10)
Indigenous females	33 (10.7%)	258 (7.5%)	62 (8.0%)	33 (4.0%)	>4
Indigenous males	41 (13.3%)	314 (9.1%)	36 (4.6%)	68 (8.3%)	<1; >3
Non-Indigenous females	113 (36.7%)	1,468 (42.5%)	451 (58.1%)	283 (34.5%)	<3; >4
Non-Indigenous males	121 (39.3%)	1,414 (40.9%)	227 (29.3%)	437 (53.2%)	>3; <4
Place of residence					
Missing [n, (%)]	28 (9.1%)	154 (4.5%)	12 (1.6%)	316 (38.5%)	960.27*** (.42) >2; >3; <4
Metropolitan^†^ [n, (%)]	159 (56.8%)	1,609 (48.8%)	406 (53.1%)	265 (52.5%)	11.28*** (.05) >2
Regional^†^ [n, (%)]	113 (40.4%)	1,537 (46.6%)	333 (43.6%)	229 (45.4%)	5.63 (.03)
Remote/very remote^†^ [n, (%)]	8 (2.7%)	154 (4.7%)	25 (3.3%)	11 (2.2%)	9.91* (.05) <2
SES^b^					
Lowest IRSAD^†^ [n, (%)]	50 (17.8%)	406 (12.3%)	74 (9.7%)	51 (10.1%)	14.79** (.06) <1
MHS contact characteristic					
Total contacts [n, (% of total contacts for sample)]	42,622 (32.3%)	24,768 (18.8%)	28,350 (21.5%)	36,290 (27.5%)	
Mean contacts per person [M, (SD)]	138.38 (113.91)	7.17 (7.48)	36.53 (29.75)	44.20 (38.87)	1598.19*** (.47) 1>2; 1>3; 1>4; 3>2; 4>2; 4>3
Age at first contact [M, (SD)]	15.54 (3.22)	16.84 (3.55)	15.22 (1.52)	11.61 (1.34)	656.75*** (.27) 1<2; 1>4; 2>3; 2>4; 3>4
Diagnosed mental disorder prevalence^c^					
Any disorder	266 (86.4%)	1,051 (30.4%)	303 (39.1%)	251 (30.6%)	402.01*** (.27) <1; <2; <4
Psychotic mental disorder	168 (54.6%)	214 (6.2%)	61 (7.9%)	25 (3.1%)	873.00*** (.40) <1; >2; >4
Mood and anxiety disorder	167 (54.2%)	534 (15.5%)	192 (24.7%)	111 (13.5%)	312.13*** (.24) <1; >2; <3; >4
Substance use disorder	151 (49.0%)	473 (13.7%)	91 (11.7%)	80 (9.7%)	311.32*** (.24) <1; >2; >4

AvePP, Average posterior probability of group classification for most likely group membership; IRSAD, Index of relative socio-economic advantage and disadvantage; OCC, Odds of correct classification; SES, socioeconomic status.

^†^Excludes cases with missing data.

<Higher than expected proportion based on *post-hoc* analysis of chi-square residuals using Bonferroni adjustment.

>Lower than expected proportion based on *post-hoc* analysis of chi-square residuals using Bonferroni adjustment.

a Percentages are column-wise, proportion within trajectory group.

b Missing data reported for *Place of residence* also applies to SES since IRSAD is calculated from location.

c Derived from hospital admissions records.

Levels of statistics significance denoted: * p <.05, ** p <.01, *** p <.001.

Tests of group differences in mean MHS contacts and mean age at first contact were conducted to confirm separation of the groups generated by the GBTM process. There was a significant difference across groups in the mean number of service contacts per individual, *F*(3,5355) = 1598.19, *p* <.001, η^2^ = .47, with all *post-hoc* trajectory group comparisons significant using Tukey multiple comparisons of means adjustment. The escalating contacts group had the highest mean number of contacts per individual, while the low contacts group had the lowest mean number of contacts per individual compared to all other groups. There was a significant difference across trajectory groups in the age at first MHS contact, *F*(3,5355) = 656.75, *p* <.001, η^2^ = .27, with all group comparisons significant except for between the escalating and adolescent limited contacts groups. The childhood peak declining contacts group were younger on average at their first contact, while the low contacts group were older on average at their first contact compared to all other groups.

**Figure 3:**
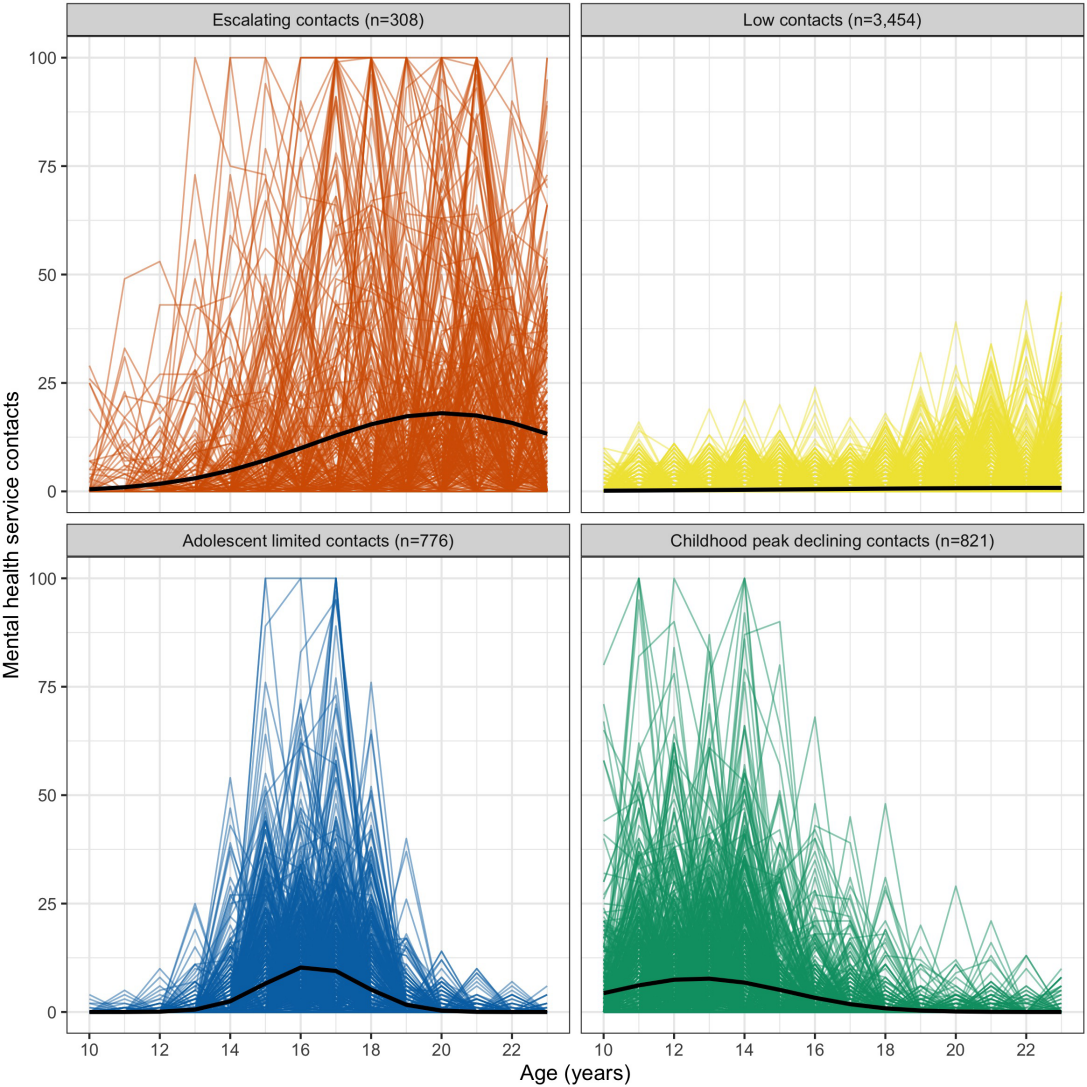
Individual trajectory profiles for MHS contacts separated by group membership.

In **Figure 3**, observed individual trajectory profiles were plotted according to group classification from the highest posterior probability of membership. Each trajectory group is represented on a separate plot, with observed individual trajectories within that group overlaid with each other. Examination of these plots highlight that although classified individual trajectories largely appeared to be consistent with the approximate shape of the fitted trajectories, there was significant variation within each group, particularly for the escalating contacts group.

### Trajectory group covariate comparisons

Finally, we examined how trajectory groups differed across covariates (see **Table 4**). The proportion of individuals assigned to groups differed significantly across the demographic intersection of sex and Indigenous status, χ^2^ (9, *N* = 5359) = 155.20, *p<*0.001, φ_c_ = .10. Indigenous males were significantly overrepresented in the escalating contacts group. Both Indigenous and non-Indigenous males were significantly under-represented in the adolescent limited contacts group; although non-Indigenous females were overrepresented in this group. Indigenous and non-Indigenous females were significantly under-represented in the childhood peak declining contacts group, but non-Indigenous males were overrepresented in this group.

Place of residence information was missing for 510 individuals and was not evenly distributed across trajectory groups, χ^2^ (3, *N* = 5359) = 960.27, *p<*0.001, φ_c_ = .42, with 38.5% of the childhood peak declining contacts group missing this information, compared to 9.1% of the escalating contacts, 4.5% of the low contacts and 1.6% of the adolescent limited contacts groups. It is possible that the higher rate of missing information for the childhood peak group may be partly explained by these being older contacts, when residence information may have been less consistently documented. This should be taken into account when interpreting results relating to place of residence (as well as SES, reported later, that was also based on location at first contact). Excluding missing cases, there were significant differences across groups in the proportion of individuals who resided in metropolitan, χ^2^ (3, *N* = 5359) = 11.28, *p<*0.01, φ_c_ = .05, and remote, χ^2^ (3, *N* = 5359) = 9.91, *p<*0.05, φ_c_ = .05, locations, but not regional locations, χ^2^ (3, *N* = 5359) = 5.63, *p* = .13, φ_c_ = .03. Adolescent limited (53.1%) and escalating (56.8%) contacts groups had the highest proportions of individuals in metropolitan residences. The low contacts group (4.7%) had the highest proportion of individuals residing in remote locations. There was a significant difference across groups in the proportion of individuals residing in locations with the lowest IRSAD value, χ^2^ (3, *N* = 5359) = 14.79, *p<*0.01, φ_c_ = .06, with more (17.8%) of the escalating contacts group residing in these locations.

There were significant differences across groups in the prevalence of diagnosed mental disorders from hospital admissions, including for any mental disorder, χ^2^ (3, *N* = 5359) = 402.01, *p<*0.001, φ_c_ = .27, psychotic mental disorders, χ^2^ (3, *N* = 5359) = 911.69, *p<*0.001, φ_c_ = .41, mood and anxiety disorders, χ^2^ (3, *N* = 5359) = 318.23, *p<*0.001, φ_c_ = .24, and substance use disorders, χ^2^ (3, *N* = 5359) = 311.32, *p<*0.001, φ_c_ = .24. The escalating contacts group had the highest proportion of individuals with any mental disorder (86.4%), psychotic mental disorders (54.6%), mood and anxiety disorders (54.2%) and substance use disorders (49.0%) compared to all other groups. Mood and anxiety disorders were the most prevalent disorders across the remaining low (15.5%), adolescent limited (24.7%), and childhood peak declining (13.5%) contacts groups. Over half of the individuals classified in the escalating contacts group had a psychotic mental disorder diagnosis, compared to between 3% to 8% of individuals in the other groups.

## Discussion

Our results demonstrate that GBTM can illustrate variability in MHS usage over the life course. Research on clinical staging models in MHSs has indicated that although some progress has been made in identifying early and subthreshold symptoms of mental disorder, a marked gap remains in determining which factors are predictive of individuals who will progress to long-term mental disorder, and time frames for these transitions (35). Addressing this gap is critical for improving the precision and timing of interventions. We identified four trajectories characterized by escalating (5.8%); low (64.5%), adolescent limited (14.5%), and childhood peak declining (15.3%) MHS contact. The low contacts trajectory contained most of the individuals (64.5%) in the sample but accounted for the lowest proportion of service contacts (18.8%). In contrast, the escalating contacts trajectory contained the fewest individuals (5.8%) but accounted for the highest proportion of contacts (32.3%). These findings demonstrate that most individuals had limited contacts with an outpatient MHS across the life-course, and that most service contacts were concentrated within a comparatively small group of individuals.

Trajectory groups varied by distribution of sex, Indigenous status, and diagnosed mental disorders. For example, the adolescent-limited trajectory group contained a higher proportion of females and higher rates of mood and anxiety disorders. This finding may reflect the higher prevalence of these mental disorders in female populations generally (36). In contrast, males were more common in the childhood peak declining trajectory group, which again, may be reflective of sex differences, with males having a greater likelihood of being referred for childhood behavioral disorders (37). This decrease in outpatient MHS contacts over time for this group may reflect individuals “aging out” of these disorders, differences in how child and adult mental health systems operate, or alternatively whether individuals are lost to MHSs due to involvement in other services/systems (e.g., youth justice services; 38).

Our results further demonstrate the importance of MHSs adopting a clinical staging framework that recognizes dynamic nature of mental disorders and intervention needs over time. For example, our findings indicate that the escalating contacts trajectory group comprises the individuals most appropriate to receive targeted early and intensive interventions to reduce the burden of enduring mental health disorders across the life course (1). This group was characterized by the highest proportion of Indigenous Australian peoples, the greatest number of contacts, and highest rates of diagnosed disorders from hospital admissions, including psychotic mental disorders and substance use disorders. Over half of the individuals in the escalating contacts group had a psychotic mental disorder compared to between 3% and 8% of individuals in the other groups. The escalating contacts group likely reflects individuals whose mental disorders emerge early in life and demonstrate a course marked by chronicity, multiple episodes of relapse, and development of comorbid mental disorders (1). This aligns with the course of some psychotic (e.g., schizophrenia) and personality (e.g., borderline personality disorder) mental disorders that typically emerge in adolescence and require increasing levels of care into early adulthood. Individuals classified into the escalating contacts group may largely represent young people who demonstrate a need for care before reaching thresholds for traditional major mental disorder diagnoses (39), highlighting the importance of targeted early intervention for this group. Targeted intervention could yield important benefits in terms of improved outcomes for highly vulnerable individuals (40) and social and economic benefit (41). However, current evidence highlights a lack of consensus regarding optimal service models for young people presenting with severe and complex mental health difficulties, with substantial variation in how clinical high-risk states and early psychosis are conceptualized and managed across services (42). Moving the field forward will require greater integration of transdiagnostic, developmentally informed, and needs-based approaches, alongside clearer care pathways that address diagnostic uncertainty, service fragmentation, and the challenges of engaging young people whose presentations often span traditional diagnostic and service boundaries (42, 43).

There are clear clinical implications of MHS utilization being concentrated in the escalating contacts group (i.e., 5.8% of individuals accounting for 32.3% of all contacts). From a service-planning perspective, this pattern suggests that relatively modest improvements in early identification, engagement, and continuity of care for this subgroup could yield disproportionate benefits for both consumer outcomes and system capacity. These findings support a stratified model of care in which intensity and duration of follow-up are matched to predicted service-use trajectories, including caseload weighting and proactive allocation of multidisciplinary resources (e.g., assertive outreach, senior clinical oversight, integrated substance use and psychosis capability) for young people at highest risk of chronicity and repeated episodes (43).

The trajectory patterns suggest specific opportunities for early intervention. First, group differences in age at first contact indicate that the escalating subgroup is visible within services by mid-adolescence, providing a window for enhanced assessment and stepped intensification of care before patterns of chronic high utilization become established. Second, the population-level peaks in contacts around late adolescence and early adulthood, combined with an apparent dip between these periods, are consistent with vulnerabilities associated with transitions between youth and adult services. Service redesign efforts that priorities continuity, including shared-care or “bridging” models, active transfer protocols, and rapid re-engagement pathways, may mitigate disengagement and relapse during this transition (43).

Although trajectory membership cannot be known at the point of first presentation, routinely captured administrative indicators could support prospective risk identification and stratification. For example, a high frequency of contacts in the initial period of care, early inpatient admissions (particularly for psychotic or comorbid substance-related disorders) and contextual disadvantage (e.g., residing in low SES location) may identify young people who would benefit from earlier assertive follow-up and integrated, multidisciplinary treatment. Future work could test prediction models that use these routinely collected signals to guide stepped-care decisions and ensure timely escalation to more intensive or specialized services.

The higher proportion of Indigenous Australian individuals in the escalating group highlights the importance of culturally responsive and appropriate approaches in early and targeted modes of intervention. Notably, sex and Indigenous status differences emerged in the escalating group with Indigenous females experiencing the highest levels of service contact, peaking at age 16. It has been estimated that Indigenous Australians, who now account for approximately 3.3% of the overall Australian population (44), experience a burden of disease and injury 2.3 times that of non-Indigenous Australians (45). Mental and substance use disorders account for the greatest proportion of this disease burden for Indigenous peoples (45). In Australia, access to health services by Indigenous populations is influenced by multiple factors. These include acceptability, perceived effectiveness, availability, affordability, travel dynamics, and social and cultural considerations (46). Page et al. (10) state that to reduce the gap between Indigenous and non-Indigenous Australians, improved planning and service provision, and Indigenous-specific models of mental health care are needed (e.g., 47).

The escalating contacts group had a comparatively higher proportion of individuals from low SES locations. It is well established that socioeconomic disadvantage is associated with an increased vulnerability to the developmental of mental health problems (48). MHSs therefore need to recognize the wider difficulties that individuals are likely to face (e.g., poor housing, unemployment) that may impact the effective delivery of interventions. In this context, it is likely that MHSs could be strengthened by the integrated delivery of social services to address broader needs (e.g., 40).

The adolescent limited contacts group showed an increase in mental disorder service contacts following the onset of adolescence, with contacts declining in early adulthood. This pattern may reflect aging out of adolescent mental disorder. However, adolescent limited contacts may also reflect a treatment discontinuation due to lack of continuity between youth MHSs and their adult service counterparts (49). Given that transitioning to adult MHSs is seen as a point of vulnerability for young people, these results contribute to investigations regarding how services can offer better support during transition periods. It should be noted that our study data is limited to age 23, and thus may be an artificial representation of cessation for some adolescents.

Taken together, our results demonstrate that a small proportion of individuals who experience serious mental disorders will require intensive and prolonged MHS contact. Identifying these individuals early in their treatment pathways, and then providing early intervention for their emerging mental disorder, has the potential to reduce the need for high levels of ongoing service contact over the life course. Identification of location and timing of first service contacts, and referral pathways or opportunities for these individuals creates opportunities for early identification and intervention, potentially resulting in considerable individual and social economic benefit.

The results of this study should be interpreted with consideration of the strengths and limitations of the data and methodology. Population-based, longitudinal studies provide policy makers and planners with vital reliable and valid information to guide the development of strategies to effectively manage individuals who have contact with MHSs and experience mental disorder. The use of a birth cohort allowed us to examine variations in relation to sex, Indigenous status, SES, and place of residence. Using the GBTM methodology we were able to model the longitudinal nature and intensity of MHS contacts more extensively. Despite these strengths, there are also challenges in using these data as they can potentially be impacted by diagnostic or administrative error. Further, availability of administrative data pertaining to diagnosed mental disorder and service contacts depends in part on the availability of services, which will vary across jurisdictions, such as rural and remote areas (50). Our study did not control for some known risk factors that could impact mental disorder and MHS system contacts, including unemployment, relationship status, and morbidity. The use of hospital admissions and community MHS contacts to identify mental disorder does not capture the full extent of psychiatric morbidity, given these admissions may be biased toward more severe and/or acute phases of psychiatric disorders or distinct population subgroups who are able to access services. Therefore, the extent of mental disorder reported in this study is conservative. Finally, we only included records from one Australian jurisdiction, meaning service contacts occurring interstate or overseas are not captured. Regardless, many of these limitations are common to studies using administrative data more generally, and do not negate the findings of the current research.

## Conclusion

We adopted a novel methodological approach to advance understanding of life-course variations in MHS contacts. We identified four distinct trajectories of service contact, characterized by important differences in diagnoses, sex, Indigenous status, SES, and location. In doing so, we demonstrated the value of this methodological approach for improving understanding of variations in access, vulnerability, and opportunities for intervention. Most notably, we identified a trajectory (escalating contacts) that appears to consist of individuals most appropriate to receive targeted early and intensive interventions (1). Future research could expand on this work by further exploring individual characteristics, precursors, and pathways into initial mental health system contacts. Efficacious intervention with this small but distinct group has the potential to meaningfully impact available system resources and improve outcomes for these individuals.

## Data Availability

The data for the study are held in Social Analytics Lab (SAL) at Griffith University. Due to privacy, ethical and legal considerations, the Queensland Cross-sector Research Collaboration data cannot be shared without direct approval from relevant data custodians and Queensland Government Statistician’s Office. Any researcher interested in accessing the data can submit an application to the SAL management committee (socialanalyticslab@griffith.edu.au) with the relevant support and approvals. Requests to access these datasets should be directed to socialanalyticslab@griffith.edu.au.

## Footnotes

1 In this article, we respectfully refer to Aboriginal and Torres Strait Islander Peoples as Indigenous Australians or Indigenous peoples. We acknowledge that the overarching term of Indigenous status is problematic, since it obscures the diversity of the more than 500 Aboriginal and Torres Strait Islander nations in Australia. However, the use of this overarching term is unavoidable in our analyses, as the QCRC datasets do not provide further detail on identity.

2 The Australian State of Queensland is characterised by significant geographic diversity in access to services. Remoteness areas divide locations into five classes of remoteness based on a measure of relative geographic access to services. For the current analysis, we combined inner and outer regional, and remote and very remote location classes.
